# Comparison of health-related quality of Life (HRQOL) among patients with pre-diabetes, diabetes and normal glucose tolerance, using the 15D-HRQOL questionnaire in Greece: the DEPLAN study

**DOI:** 10.1186/s12902-018-0261-3

**Published:** 2018-05-29

**Authors:** Konstantinos Makrilakis, Stavros Liatis, Afroditi Tsiakou, Chryssoula Stathi, Eleftheria Papachristoforou, Despoina Perrea, Nicholas Katsilambros, Nikolaos Kontodimopoulos, Dimitrios Niakas

**Affiliations:** 1 First Department of Propaedeutic Medicine, National and Kapodistrian University of Athens Medical School, Laiko General Hospital, 17 Ag. Thoma St, 11527 Athens, Greece; 2 Laboratory for Experimental Surgery and Surgical Research “Christeas Hall”, University of Athens Medical School, Athens, Greece; 3 Hellenic Open University, Patras, Greece

**Keywords:** Diabetes mellitus, Pre-diabetes, Quality of life, HRQOL-15D questionnaire

## Abstract

**Background:**

Diabetes mellitus is usually preceded by a pre-diabetic stage before the clinical presentation of the disease, the influence of which on persons’ quality of life is not adequately elucidated. The purpose of this study was to compare the Health-Related Quality of Life (HRQOL) of persons with pre-diabetes with that of diabetes or normal glucose tolerance (NGT), using the validated HRQOL-15D questionnaire.

**Methods:**

The HRQOL-15D scores of 172 people with pre-diabetes (108 with Impaired Fasting Glucose [IFG], 64 with Impaired Glucose Tolerance [IGT], aged 58.3 ± 10.3 years) and 198 with NGT (aged 54.4 ± 10.1 years) from the Greek part of the DEPLAN study (Diabetes in Europe - Prevention using Lifestyle, Physical Activity and Nutritional Intervention), were compared to 100 diabetes patients’ scores (aged 60.9 ± 12.5 years, diabetes duration 17.0 ± 10.0 years, HbA1c 7.2 ± 1.2%), derived from the outpatient Diabetes Clinic of a University Hospital.

**Results:**

The diabetes patients’ HRQOL-15D score (0.8605) was significantly lower than the pre-diabetes’ (0.9008) and the controls’ (0.9092) (*p* < 0.001). There were no differences in the total score between the controls and the group with pre-diabetes. However, examination of individual parameters of the score showed that people with IGT had lower scores compared to the control group, as related to the parameters of “mobility” and “psychological distress”. No differences were found in any component of the HRQOL-15D score between the control group and the IFG group, nor between the two groups with pre-diabetes (IFG vs. IGT).

**Conclusions:**

Persons with pre-diabetes had a similar HRQOL score with healthy individuals, and a higher score than persons with diabetes. Specific components of the score, however, were lower in the IGT group compared to the controls. These findings help clarify the issue of HRQOL of persons with pre-diabetes and its possible impact on prevention.

## Background

Diabetes mellitus (DM) is a chronic disease with serious complications, imposing a significant burden on the health status of affected individuals, both on physical and mental aspects [1, 2]. Its commonest form, Type 2 DM (T2D), usually follows distinct stages in its development: from normal glucose tolerance (NGT), to impaired glucose metabolism (pre-diabetes), and overt onset of the disease [3]. It is well established that the quality of life (QOL) of people with diabetes (total physical, mental, and social well-being) is adversely affected by the disease and its complications [4]. Concerning the QOL of persons with pre-diabetes, however, there is sparse and controversial data in the literature [5–8], possibly related to different methods of health-related QOL (HRQOL) measurement, small sample sizes or focus on selected populations (for example, elderly, instead of the general population) [9]. Especially in the Greek population, to our knowledge, no data exist at all on this matter.

Although people with pre-diabetes experience no symptoms and usually have no knowledge of their condition [10], there is evidence that around 10–20% of them already have some mild micro- or macro- vascular complications [11], which might confer some adverse impact on their HRQOL, or at least in some aspects of it [12]. The prevalence of DM in Greece remains high, and according to recent data [13] it accounts for 7.0% of the population (with 8.2% prevalence of T2D for people ≥15 years of age). On the other hand, pre-diabetes prevalence is not well studied, with some estimates from regional studies raising it to around 22% of the adult population [14].

The DEPLAN study (Diabetes in Europe - Prevention using Lifestyle, Physical Activity and Nutritional Intervention) [15] is a European Commission-funded multinational project, aiming to establish a model for the efficient identification of individuals at high risk for T2D in the community, in the primary care structure, in the EU member countries and to test the feasibility and cost-effectiveness of the translation of the intervention concepts learned from the prevention trials into existing health-care systems [16]. Data on the quality of life of subjects with pre-diabetes and NGT from the Greek part of this study [14, 17], based on the validated health-related quality of life [HRQOL]-15D questionnaire [18], were compared to respective data of patients with diabetes, derived from the outpatient Diabetes Clinic of the “Laiko” University Hospital, in Athens, Greece, in an effort to elucidate if any differences exist in the HRQOL among these groups.

## Methods

### Participants

The sample population of the present cross-sectional study consisted of persons with pre-diabetes (Impaired Fasting Glucose [IFG], Impaired Glucose Tolerance [IGT] or both) and people with NGT (that had provided data on their HRQOL in the Greek part of the DEPLAN study), as well as persons with known DM from the outpatient Diabetes Center of “Laiko” University hospital, in Athens, Greece. This study has been previously described in detail [14, 17]. In brief, the FINDRISC questionnaire [19] was distributed to around 7900 persons without known diabetes, aged 35–75 years, residing in the metropolitan area around Athens, in order to find people at high risk for developing T2D (a score ≥ 15 signifying high probability). Out of the 3240 completed questionnaires, 869 persons accepted to undergo an oral glucose tolerance test (OGTT), so as to identify people with unknown (screen-detected) diabetes and exclude them from further intervention. On the day of the OGTT, weight, height, waist circumference and blood pressure of the participants were measured and their medical histories recorded. Presence of co-morbidities (defined as hypertension and/or dyslipidemia) and vascular complications (any combination of coronary heart disease, stroke, peripheral arterial disease, nephropathy, retinopathy or neuropathy) were also recorded. Plasma glucose, total- and high density lipoprotein (HDL)-cholesterol and triglyceride levels were measured from fasting blood samples at a central accredited university research laboratory, using enzymatic assays. Low density lipoprotein (LDL)-cholesterol was calculated using the Friedewald formula [20].

According to the OGTT results, subjects were categorized as having normal glucose tolerance (NGT), impaired fasting glucose (IFG), impaired glucose tolerance (IGT) or diabetes. IFG was defined based on a fasting plasma glucose of 100–125 mg/dl, IGT as a 2-h plasma glucose between 140 and 199 mg/dl and (screen-detected) DM as a fasting plasma glucose ≥126 mg/dl and/or 2-h plasma glucose ≥200 mg/dl [3]. People with both IFG and IGT were considered as IGT. Persons with screen-detected DM from the DEPLAN cohort were not included in the present analysis. These people did not know they had DM before performing the OGTT and were thus thought they represented a special category of patients with diabetes (newly diagnosed), resembling more to the pre-diabetes group as regards to complications and QOL issues. The HRQOL data of the persons with pre-diabetes and the controls from the DEPLAN cohort were compared to respective data of people with known diabetes, derived from the outpatient Diabetes Center of “Laiko” University hospital.

The participants’ HRQOL was recorded using the 15D questionnaire [18], a preference-based HRQOL instrument that has also been validated in the Greek population [21]. The reason that this measure was used in the present study is that this is the HRQOL instrument that had already been used in the DEPLAN study where the participants with pre-diabetes and NGT were derived from. License to use this HRQOL questionnaire had been centrally obtained from the Steering Committee of the original European DE-PLAN study and was used by all participating centers [15]. No other QOL measurements were available for the DEPLAN participants. The 15D-questionnaire contains 15 dimensions (questions): mobility, vision, hearing, breathing, sleeping, eating, speech, excretion, usual activities, mental function, discomfort and symptoms, depression, distress, vitality and sexual activity, each having five different levels of functional status. These dimensions can be presented as a 15-dimensional profile or as a one-index score. The 15D index score is obtained by weighing the dimensions with population-based preference weights based on an application of the multi-attribute utility theory. Obtained index scores vary between 0 and 1, where 0 represents a state of being dead and 1 represents perfect HRQOL [22]. Questionnaires were distributed to the participants and were self-filled, blindly to the investigators.

The study was approved by the cooperating hospital’s ethics committee (Laiko General Hospital Ethics Review Board), and the Hellenic National Drug Organization. All participants signed an informed consent according to the general recommendations of the Declaration of Helsinki [23].

### Statistical analysis

Continuous variables are presented as mean ± one-standard deviation, while qualitative variables as absolute and relative frequencies (%). Normal distribution of variables was tested with the Shapiro-Wilk test. Comparisons between 2 normally distributed continuous variables were performed with the calculation of the Student’s t-test, whereas the Wilcoxon Mann-Whitney U-test was used for non-parametric variables. Associations between categorical variables were tested with the use of contingency tables and the calculation of the Chi-squared test. Pearson’s correlation coefficient (r) or Spearman’s rho (for non-normal distributions) were used for the evaluation of statistical correlations between variables. For comparisons of ≥3 variables, one-way analysis of variance (ANOVA) (for normally distributed variables), or the Kruskal-Wallis test (for non-normally distributed variables) was used. For controlling of confounding variables (such as age, gender, smoking, body mass index [BMI], hypertension, complications, co-morbidities) analysis of covariance (ANCOVA) was used. All reported *p*-values are derived from two-sided tests and compared to a significance level of 5%. Data were analyzed using the Statistical Package SPSS, version 23.0 (SPSS Inc., Chicago, IL).

## Results

Out of the total 869 persons screened with an OGTT in the DEPLAN cohort, 383 (44.1%) had complete HRQOL data. The present analysis included 370 participants (mean age [±SD] 57.2 ± 11.0 years, 46% males), out of whom 172 had pre-diabetes (108 with IFG, 64 with IGT, aged 58.3 ± 10.3 years) and 198 had NGT (aged 54.4 ± 10.1 years). Thirteen individuals (age 64.2 ± 4.1 years, BMI 30.4 ± 6.4 kg/m^2^) had screen-detected diabetes and, as explained above, due to their recent diagnosis and small number, precluding any meaningful statistical analysis as a separate group, were excluded from further analysis. The diabetes group in the present analysis was comprised of 100 persons (mean age 60.9 ± 12.5 years, DM duration 17.0 ± 10.0 years, HbA1c: 7.2 ± 1.2%) from the outpatient Diabetes Center of “Laiko” University hospital.

The demographic, clinical and laboratory characteristics of the study participants are presented in Table 1. As shown, people with diabetes were older, mostly males (59%), smoked less and had more frequently co-morbidities and vascular complications than the other two groups. Of note, individuals with pre-diabetes were more obese than the other two groups and had more co-morbidities than the NGT group (48.8% vs. 35.2%, respectively, *p* = 0.008), but the frequency of vascular complications did not differ between them (11.9% vs. 8.2%, respectively, *p* > 0.05).

**Table 1:** Demographic, clinical and laboratory characteristics of participants (mean ± SD)

Variable	NGT	Pre-Diabetes			DM	P*
		IFG	IGT	All Pre-DM		
Number	198	108	64	172	100	–
Gender (male) [n (%)]	74 (37.4)	59 (54.6)	25 (39.1)	84 (48.8)	59 (59.0)	0.001
Age (years)	54.4 (10.1)	57.2 (10.1)	60.3 (10.5)	58.3 (10.3)	60.9 (12.5)	< 0.001
Weight (kg)	81.1 (15.6)	88.6 (13.6)	87.2 (14.7)	88.1 (14.0)	85.2 (20.4)	0.001
BMI (kg/m^2^)	29.4 (5.3)	31.5 (4.3)	32.2 (5.4)	31.7 (4.8)	29.6 (6.5)	< 0.001
Smoking (%)	56.6	58.3	53.1	56.4	37.1	0.007
Co-morbidities [n (%)]	69 (35.2)	48 (44.4)	36 (56.3)	84 (48.8)	74 (87.1)	< 0.001
Complications [n (%)]	5 (8.2)	7 (9.1)	8 (16.3)	15 (11.9)	26 (30.6)	< 0.001
SBP (mmHg)	119.3 (18.5)	129.5 (16.2)	128.0 (16.1)	128.9 (16.1)	134.9 (18.9)	< 0.001
DBP (mmHg)	75.9 (12.0)	78.9 (11.5)	77.4 (11.5)	78.3 (11.5)	74.9 (10.4)	NS
Cholesterol (mmol/L)	5.47 (0.97)	5.70 (0.97)	5.82 (1.01)	5.75 (0.99)	4.24 (1.08)	< 0.001
Triglycerides (mmol/L)	1.18 (0.57)	1.49 (0.88)	1.57 (0.71)	1.52 (0.36)	1.43 (0.74)	< 0.001
HDL-C (mmol/L)	1.20 (0.21)	1.22 (0.23)	1.25 (0.19)	1.23 (0.22)	1.22 (0.28)	NS
LDL-C (mmol/L)	3.72 (0.88)	3.82 (0.84)	3.86 (0.94)	3.84 (0.88)	2.35 (0.91)	< 0.001
DM duration (years)	–	–	–	–	17.0 (10.0)	–
HbA1c (%)	–	–	–	–	7.2 (1.2)	–

*NGT* Normal Glucose Tolerance, *DM* Diabetes mellitus, *SBP* Systolic blood pressure, *DBP* Diastolic blood pressure, *BMI* Body mass index, *NS* Non- significant, *Co-morbidities* Hypertension and/or dyslipidemia, *Complications* Any combination of coronary heart disease, stroke, peripheral arterial disease, nephropathy, retinopathy, neuropathy

*P = Comparison among the 4 groups (NGT, IFG, IGT, DM) by Chi-squared or Kruskal-Wallis analysis

Simple correlation analyses showed that the HRQOL-15D score was negatively correlated with age (Spearman’s rho = − 0.13, *p* = 0.010), HDL-cholesterol (rho = − 0.11, *p* = 0.030), and BMI (rho = − 0.14, *p* = 0.004), and positively with LDL-cholesterol (rho = 0.10, *p* = 0.050). Specifically, within the group of patients with diabetes, there was a negative correlation of the HRQOL-15D score with DM duration (rho = − 0.34, *p* = 0.001) and a trend for a negative correlation with glycemic control (as measured by HbA1c) (rho = − 0.20, *p* = 0.058).

**Fig. 1:**
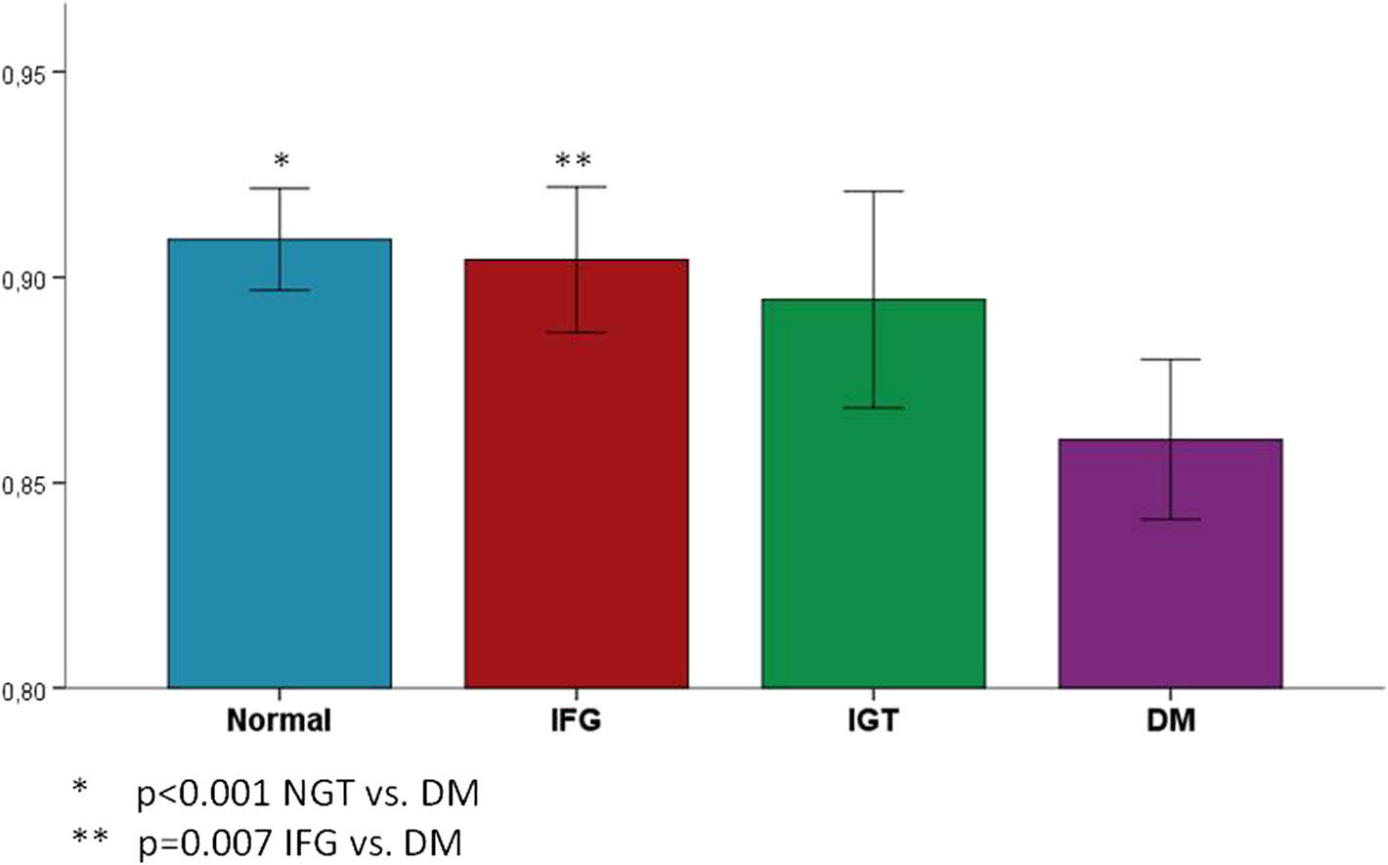
HRQOL-15D scores in NGT, pre-diabetes (IFG-IGT) and diabetes persons

Table 2 shows the results of the comparison of the HRQOL-15D score (and its components) among the groups of NGT, pre-diabetes (IFG – IGT) and DM participants. Patients with diabetes had a lower total HRQOL-15D sore (0.8605) compared to the other two groups (0.9092 and 0.9008, for the NGT and pre-DM group, respectively, *p* < 0.001 by Kruskal-Wallis analysis), while IFG and IGT participants had similar scores (0.9043 and 0.8946, respectively). In *post-hoc* analyses, it was shown that there was a significant difference between the group of patients with diabetes and the NGT group (*p* < 0.001) as well as between the diabetes and the IFG group (*p* = 0.007). On the contrary, there were no statistically significant differences in the HRQOL score between any two of these three groups (NGT, IFG and IGT) (Fig. 1).

**Table 2:** Comparison of the HRQOL-15D score and its components among the DM patients, people with pre-DM (IFG – IGT) and NGT

	NGT	Pre-diabetes			DM	P*
		IFG	IGT	All pre-DM		
Mobility	0.9179	0.9122	0.8711	0.8969	0.8264	< 0.001
Vision	0.8688	0.8963	0.8938	0.8954	0.8333	NS
Hearing	0.9487	0.9562	0.9455	0.9522	0.9152	NS
Breathing	0.9150	0.8849	0.8862	0.8854	0.8473	0.044
Sleeping	0.8335	0.8385	0.8256	0.8338	0.8172	NS
Eating	0.9983	1.0000	0.9945	0.9980	0.9901	NS
Speech	0.9887	0.9880	0.9844	0.9867	0.9676	NS
Excretion	0.9433	0.9511	0.9234	0.9410	0.9048	NS
Usual activities	0.9214	0.9329	0.8956	0.9191	0.8226	< 0.001
Mental function	0.9153	0.9095	0.9068	0.9085	0.9007	NS
Discomfort and symptoms	0.8841	0.8779	0.8683	0.8743	0.8694	NS
Depression	0.8601	0.8627	0.8472	0.8569	0.8574	NS
Distress	0.7561	0.7333	0.6971	0.7205	0.7657	0.019
Vitality	0.8474	0.8150	0.8424	0.8246	0.8112	NS
Sexual activity	0.9000	0.8838	0.8895	0.8858	0.6642	< 0.001
Total score	0.9092	0.9043	0.8946	0.9008	0.8605	< 0.001

*NGT* Normal Glucose Tolerance, *IFG* Impaired Fasting Glucose, *IGT* Impaired Glucose Tolerance, *DM* Diabetes mellitus, *NS* Non-significant

*P = Comparison among the 4 groups (NGT, IFG, IGT, DM) by Kruskal-Wallis analysis

In a multifactorial analysis of covariance (ANCOVA), after controlling for age, gender, BMI and smoking (model 1, Table 3), the HRQOL-15D score was significantly associated with the glycemic status (NGT, pre-diabetes [IFG/IGT] or diabetes) (*p* < 0.001). Male gender (*p* < 0.001) and higher BMI (*p* = 0.003) were also significantly associated with a lower HRQOL score, and this model explained the variance of HRQOL score by 14% (R^2^ = 0.14). When the presence, however, of co-morbidities and vascular complications were added to the model (model 2, Table 4), the relationship of the glycemic status with the HRQOL-15D score was attenuated and lost significance. Male gender still had a significant contribution to the model (*p* < 0.001), whereas the independent effect of vascular complications (*p* = 0.004) negated the effects of the glycemic status and of BMI (the model now explained the overall variance of the HRQOL score by 21.8% [R^2^ = 0.218]).

**Table 3:** Analysis of covariance (ANCOVA) for the relationship between the HRQOL-15D score with glycemic status, controlling for age, gender, BMI and smoking (persons with pre-diabetes were considererd separately as IFG - IGT) (Model 1)

Variable	F	*P*
Age	0.72	NS
Gender (male)	20.05	< 0.001
BMI	8.53	0.003
Smoking (yes)	0.26	NS
Glycemic status	3.62	< 0.001

*R*^*2*^ 0.14, *BMI* Body mass index

Glycemic status: 1 = NGT, 2 = IFG, 3 = IGT, 4 = Diabetes

**Table 4:** Analysis of covariance (ANCOVA) for the relationship between the HRQOL-15D score with glycemic status, controlling for age, gender, BMI, smoking, presence of co-morbidities and vascular complications (persons with pre-diabetes were considererd separately as IFG - IGT) (Model 2)

Variable	F	*P*
Age	2.08	NS
Gender (male)	19.07	< 0,001
BMI	3.48	NS
Smoking (yes)	1.28	NS
Co-morbidities	0.37	NS
Complications	6.39	0.004
Glycemic status	0.53	NS

*R*^*2*^ 0.218, *Co-morbidities* arterial hypertension and/or dyslipidemia, *Complications* any combination of coronary heart disease, stroke, peripheral arterial disease, nephropathy, retinopathy or neuropathy

Glycemic status: 1 = NGT, 2 = IFG, 3 = IGT, 4 = Diabetes

**Fig. 2:**
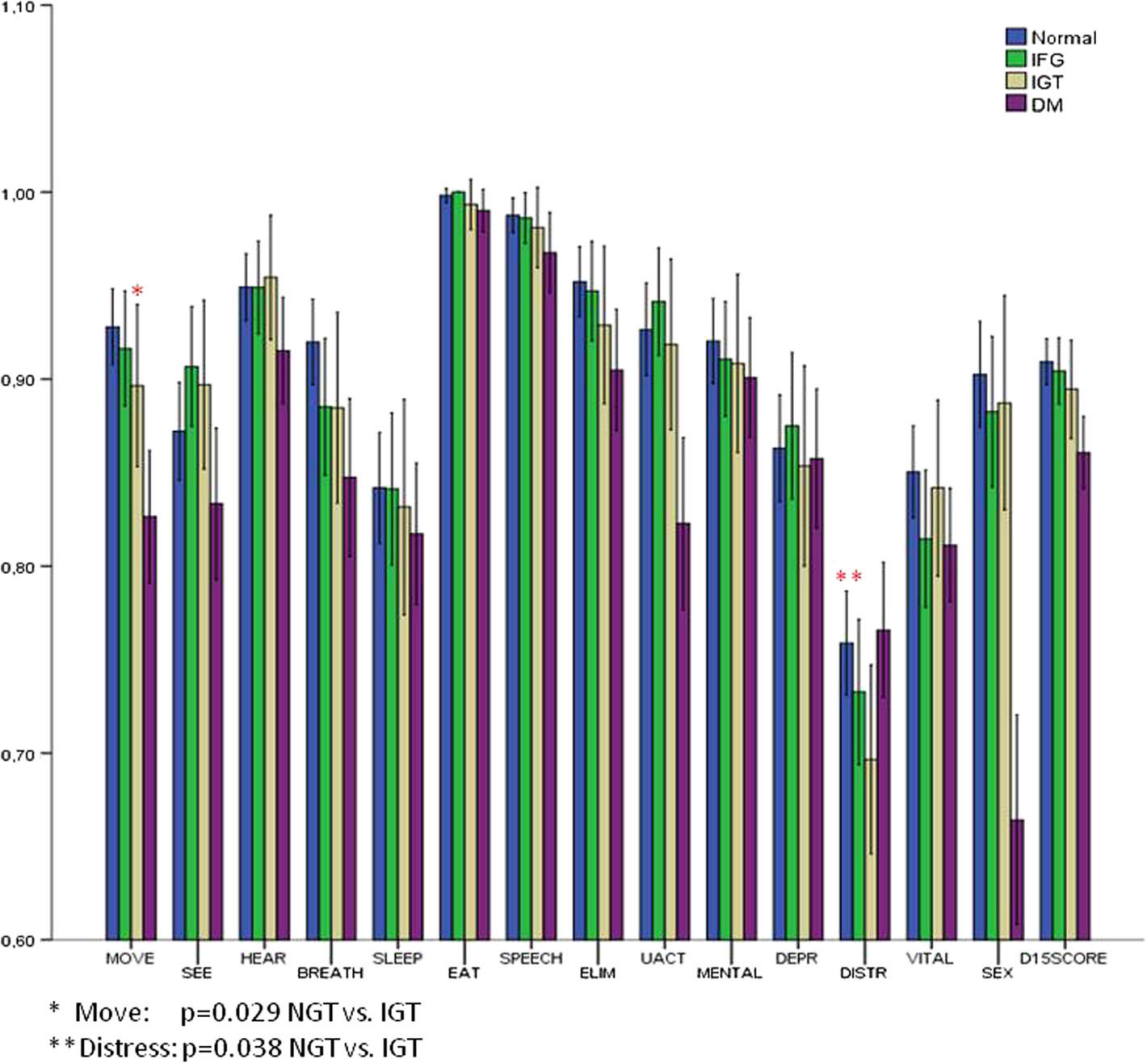
Profiles of the HRQOL-15D components among the NGT, Pre-DM (IFG – IGT) and DM participants

The different components of the HRQOL-15D score were evaluated separately among the groups. As shown in Table 2, there were statistically significant differences for the components of “mobility”, “breathing”, “usual activities”, “distress” and “sexual activity” among the groups as a whole. In *post-hoc* analyses, a statistically significant difference was found between the NGT and IGT groups as regarded to the components of “mobility” (*p* = 0.042) and “distress” (*p* = 0.01) (lower values for the IGT group), as well as between the IGT and DM groups as regarded to the components of “distress” (*p* = 0.029) (lower for the IGT group) and “sexual activity” (*p* < 0.001) (lower for the DM group). These associations were attenuated but persisted after adjustment for age, gender, BMI, presence of co-morbidities and complications. There were no differences in any component of the HRQOL-15D score between the two groups of the pre-diabetes participants (IFG and IGT), or the NGT vs. the IFG group (Fig. 2).

## Discussion

There is a lot of interest in the past few decades in studies of health-related quality of life (HRQOL) and the impact of various diseases and disease-states upon it, which has led to the development and refinement of a number of generic and disease-specific HRQOL measures [24, 25]. It should be emphasized also that clinical variables alone do not comprehensively capture patients’ perceptions of their health, which is in part due to the fact that HRQOL is influenced by many other factors, such as the existence of other health problems, social relationships, marital status, patient knowledge, treatment satisfaction and perceived ability to control one’s disease [26].

In the present study, the HRQOL of patients with diabetes was compared with that of pre-diabetes (IFG/IGT) and persons with normal glucose tolerance (NGT), using the HRQOL-15D questionnaire. It was found, that, in general, the HRQOL of patients with diabetes was significantly worse than that of the other two groups (owing mainly to the presence of vascular complications), while there were no significant differences in the overall HRQOL score between the NGT and the pre-diabetes groups. Examination, however, of the individual components of the HRQOL score showed significant differences between the NGT and the pre-diabetes group in certain aspects. In particular, the IGT group had lower scores compared to the NGT, as regarded to the components of “mobility” and “distress”. No difference was noted in any of the 15 dimensions of the score between the NGT and IFG group, nor between the two groups of the pre-diabetes subjects (IFG vs. IGT).

The deterioration of the HRQOL in people with DM [4] and the contribution of vascular complications to that effect found in the present study is in line with previous reports in the literature [27, 28]. For people with pre-diabetes, however, there are only few published studies examining the relationship of their quality of life as regards to physical [5, 12] or psychological/mental parameters [7, 8, 10], sometimes with conflicting results, either because of the use of different HRQOL measurement methods (e.g. by recording only the physical health condition and not the psychological-mental), or because of the use of small sample sizes or because of focusing on specific population groups (e.g. the elderly) [6, 9, 29]. Specifically using the HRQOL-15D questionnaire, studies in people with pre-diabetes are extremely sparse [22].

Since these people (with pre-diabetes) usually have no symptoms and no major complications and very often no knowledge of their condition [10], their HRQOL should not be expected to be affected. The fact, however, that around 10–20% of them may already have some mild micro- or macro- vascular complications [11], could explain the findings of their affected HRQOL in some aspects of it. For example, limited joint action, prayer’s sign and Dupuytren’s contracture were more common in elderly IGT persons compared to controls [12].

In the present study, ‘mobility’ was found to be impaired in the group of pre-diabetes subjects with IGT (compared to those of the control group), which is broadly in line with findings in the literature [22, 30]. It is possible that mild, even subconscious abnormalities in physical functioning could explain this finding. In a recently published prospective study [22] using 3 different assessment tools of HRQOL (SF-36, SF-6D and 15D), and dividing the subjects into 5 groups (normal glucose tolerance, IFG, IGT, newly diagnosed diabetes and known diabetes), it was found that the deterioration of the glycemic status from the stage of normal glucose tolerance to the pre-diabetes and overt diabetes was associated with a worsening of HRQOL scores, as measured with all three questionnaires. Specifically for the 15D questionnaire, decreases in the components of “mobility” (similar to the present study), “breathing”, “usual activities”, “discomfort and symptoms”, “vitality” and “sexual activity” were found, but not for the psychological dimensions of the questionnaire. These reductions - similar to the present study – did not occur in subjects with IFG but only in those with IGT or diabetes who exceeded the limits of minimal clinical significance [minimal (clinically) important differences (MIDs)] the study had set (i.e. the smallest change a patient or health professional can notice - for the 15D questionnaire MID was proposed at ≥0.02–0.03 units of the total score). A similar population study from Spain (Di@bet.es Study) [30], in 5047 individuals of the general population, using the SF-12 questionnaire, showed that women had worsening quality of life scores (relating both to physical and psychological parameters) with the deterioration of the glycemic status towards the pre-diabetes and diabetes states, while in men only physical parameters were affected (similarly in the present study male gender was independently associated with worsening HRQOL).

Other population studies from Australia (AusDiab study), using the quality of life short form-36 (SF-36) questionnaire, showed that people with IFG (especially women) [5] or IGT [31], had reduced values in mainly physical dimensions of quality of life, especially bodily pain and physical functioning, and in general health status [32]. On the contrary, in a population study in Western Finland (the Harmonica Project) in 1383 subjects, aged 45–70 years, no differences in HRQOL were detected (with the same questionnaire SF-36) in participants with pre-diabetes compared with non-diabetes subjects [6]. In this study, people with known cardiovascular disease were excluded in advance, which limits the generalization and validity of the results. In the largest population study to date [8], that included 55,882 people of the general population in Sweden (Västerbotten Intervention Program), using the Health Utility Weight [HUW] SF-6D questionnaire (that included the dimensions of physical functioning, role limitations, social function, bodily pain, mental health, and vitality), there was also a gradual decrease in HUWs with a progressive deterioration of the glycemic status from normal glucose tolerance to pre-diabetes and overt diabetes.

Another significant finding in the present study was that the “psychological distress” appeared to be highly affected in the group of pre-diabetes individuals with IGT (relative to normal, and surprisingly even to people with diabetes). Of note, the recording of this fact in the HRQOL-15D questionnaires was done before the participants were informed about the results of the OGTT tests that they belonged to the pre-diabetes group. Several studies in the literature have reported worsening of the psychological state in people with diabetes [2, 33], which may be caused by the impact of the diagnosis of diabetes itself, the psychological stress associated with the management of diabetes or the burden of diabetic complications [34], or even through physiological pathways, including inflammatory processes and reductions in neurotrophic function [35], which in turn may lead to reduced plasticity of neuronal networks and subsequently depression [36]. For pre-diabetes, however, the correlations that have been found are less robust. In initial studies, it was observed that depressive symptoms were more frequent in women with pre-diabetes [37], but a recent meta-analysis concluded that the risk for depression was not increased in impaired glucose metabolism compared to normal glucose metabolism or even undiagnosed diabetes subjects [38]. In the present study, “depression” did not differ between the groups of NGT, pre-diabetes or diabetes subjects.

The relationship between mental disorder and the affected glucose metabolism is likely to be bidirectional, as depressive symptoms or psychological distress may also lead to a higher risk of developing pre-diabetes (especially in men) [39] or diabetes [40]. Higher work distress has also been associated with prevalent diabetes and especially pre-diabetes in a German cohort, especially in men [41], which could also explain the findings of increased “distress” of participants with pre-diabetes in the present study, although no etiology of distress (e.g. work-related, social, family, etc) was elucidated.

There are several limitations of the present study. They include the relatively small sample size examined and the fact that it is a cross-sectional study, and thus cannot demonstrate cause and effect or the time frame in which indices of the HRQOL deteriorate. For this purpose, prospective studies are required, with a significant population sample and sufficient monitoring time. In such a relatively small study from Germany [7], there was a trend for a decline in the quality of life (only for physical parameters, as measured by the SF-12 questionnaire) within 7 years from the transition of NGT to pre-diabetes, but the association was statistically significant only for the subjects converting from NGT to diabetes.

Another limitation of this study is that the population examined is not necessarily representative of the general population, since the participants without diabetes selected themselves to participate in the study, while people with diabetes were derived from a large Diabetes University Center (Laiko Hospital), and thus the findings are not necessarily applicable to the general population. Also, the fact that the HRQOL-15D questionnaire is not specific for diabetes [25], may probably have as a result that the responses to it reflect problems associated with other conditions. The fact that it was applied only once may additionally preclude its ability to find fluctuations of HRQOL over time.

It has to be emphasized also, that there were many missing data regarding presence of vascular complications in the group of individuals with pre-diabetes (46 persons) and NGT (137 persons), which may have influenced the aforementioned comparisons.

On the other hand, strengths of the present study include the fact that the determination of the glycemic status was performed with a glucose tolerance test (OGTT) and was not self-reported, which enhances the reliability of the reported correlations. Also the HRQOL-15D questionnaire was completed by the participants of the DEPLAN cohort before they had learned the results of the OGTT, and thus their answers were not affected by the knowledge of their glycemic status. In addition, in a comparative evaluation of the HRQOL-15D questionnaire with other HRQOL assessment questionnaires in the Greek population [42], the 15D was found to be superior as regards to the assessment of vascular complications in diabetes (particularly for coronary heart disease and diabetic retinopathy). Furthermore, the exclusion of the few newly diagnosed (screen-detected) people with diabetes from the analysis, whose participation could cause distortion of the associations found, because of their actual position in-between the states of pre-diabetes and diabetes strengthens the findings of the study.

## Conclusions

In conclusion, the quality of life of individuals with pre-diabetes was overall not significantly different from that of normal glucose tolerance subjects, whereas for participants with diabetes it was lower (mainly due to the presence of vascular complications). However, certain components of the quality of life were already affected in the pre-diabetic state of IGT (compared to the control group), specifically “mobility” and “psychological distress”. Providing an understanding of the stages of diabetes where health status is diminished will allow prioritization of intervention efforts, and enable more effective targeting of policy and strategic interventions to improve health outcomes. Thus, quality of life issues (in particular physical and psychological-emotional issues) should be investigated when people with pre-diabetes are diagnosed in every-day routine clinical practice, since their identification could potentially lead to more effective overall management of their condition.

## Abbreviations

DM: Diabetes mellitus
HRQOL: Health related quality of life
IFG: Impaired fasting glucose
IGT: Impaired glucose tolerance
